# Microwave-Assisted Extraction Coupled with Mass Spectrometry for Determining Five Volatile Compounds from Soy Sauces

**DOI:** 10.1155/2021/6625929

**Published:** 2021-04-05

**Authors:** Kai Xu, Xun Gao, Miaomiao Chi, Kexin Chen, Yue Zhang, Weihao Kong, Ziying Li, Shengnan Huang, Kunming Qin

**Affiliations:** ^1^Jiangsu Key Laboratory of Marine Pharmaceutical Compound Screening, Jiangsu Ocean University, 59Cangwu Road, Lianyungang 222005, China; ^2^Jiangsu Institute of Marine Resources Development, 59 Cangwu Road, Lianyungang 222005, China; ^3^Co-Innovation Center of Jiangsu Marine Bio-industry Technology, Jiangsu Ocean University, Lianyungang 222005, China

## Abstract

As a popular fermented condiment in oriental countries, soy sauce plays a more and more important role in modern food culture due to its unique smell and delicious taste. With the help of microwave extraction and gas chromatography-tandem mass spectrometry, the sample preparation method is aimed to determine the content of cyclohexane, benzene, toluene, chlorobenzene, and styrene in soy sauce. The method was validated by examining the linearity, accuracy, specificity, precision, the limit of detection, and quantitation. Meanwhile, three key factors have an impact on the efficiency and accuracy of the method including extracting solvent, temperature, and time which were optimized. The result shows that the recoveries of spiked analytes ranged from 80.86% to 105.71%, the relative standard deviation of intraday and interday precision was no more than 12.1% and 12.5%, and the limit of detection and quantitation were 0.25–1.00 ng/mL and 0.50–2.00 ng/mL, respectively. The results also indicated that the proposed method was a simple, reliable, and sensitive approach for the determination trace amount of five harmful volatile organic compounds from soy sauce.

## 1. Introduction

Soy sauce has been widely used as a seasoning or condiment in eastern Asia and Western countries, due to its unique taste and aroma [1]. In addition, soy sauce could also provide an effective strategy to prevent micronutrient deficiency [2]. The previous reports revealed that there were various volatile organic compounds in soy sauce. For example, deoxynivalenol (DON) in soy sauce was investigated, and the results suggested that the risk of exposure to DON from the consumption of soy sauce was very low for the residents in China [3]. Several researchers reported that alcohols and acids are the main volatiles in soy sauce, while esters, furans, and phenols are part of the total volatile substances [4, 5]. Most of the studies focused on the volatile compounds related to the flavor in soy sauce, such as the fact that how 2-methyl-3-furanthiol in fermented soy sauce contributed to the meat-like aroma [6] and the fruit-like aroma in soy sauce is due to the degradation of five ethyl esters during heating [7]. Due to the wide distribution of manufacturers and varied manufacturing processes, some of the volatile organic compounds may be generated from the packaging materials.

There are many types of packagings for soy sauce, and in recent years, plastic has been widely used due to its low cost, flexibility for shaping, and comparatively lighter weight. However, with the continuous increased usage of plastic materials, food safety problems have become a significant issue. The pollution of plastics mainly comes from two aspects. On the one hand, due to the complexity of the raw materials and processes in the production of plastics, plastic packaging materials inevitably contain some small-molecule chemical substances, including processing raw materials, additives, monomers, and oligomers. On the other hand, some small-molecule chemical substances are unintentionally introduced into the plastic during the production and regeneration process, such as degradation products of raw materials and residue solvents. Soy sauce may be contaminated by some volatile organic compounds from plastic packaging materials during production, such as cyclohexane, benzene, toluene, chlorobenzene, and styrene, the amount of which will rise with the increased temperature and contacting time. When unwanted compounds exceed a certain level, they will harm people's health. Therefore, member countries of the Food and Agriculture Organization of the United Nations and World Health Organization Codex Committee are considering establishing guidelines for safety and quality control for the soy sauce products [8]. However, there are few reports about the five volatile organic compounds in soy sauce. Therefore, the research aims to develop a simple and effective method for the determination of the above-five volatile organic compounds in soy sauce, which may contribute to food safety and quality control of soy sauce.

GC-MS plays an important role in many analytical areas. Due to its high sensitivity, excellent separation performance, and low sample injection volume/diagram, GC-MS is especially necessary for the detection of multicomponents. Before analyzing by GC-MS, the sample preparation process is essential, and it also affects the accuracy of the results to a certain extent. With the continuous advancement of science and technology, diverse sample preparation methods are developed and reported, which can meet the requirements of samples with different characteristics. For volatile organic compounds in soy sauce, dynamic headspace [9, 10], direct solvent extraction [11, 12], vacuum simultaneous steam distill-solvent extraction [13], and solid-phase microextraction [14–16] have been reported, and headspace solid-phase microextraction has been used by more and more researchers due to its efficiency and accuracy [17–20]. However, these methods, which are widely used in food extraction, have shortcomings such as the fact that they are time-consuming and complex in the operation.

Microwave extraction (ME) is a new technology for extracting samples using microwave energy. Compared with the traditional heating method via conduction, the principle of using microwaves to enhance the extraction efficiency of volatile organic compounds is that, under the action of the microwave field, ions flow in a directional flow to form an electric current, which rub and collide with the surrounding molecules and other flowing ions so that the microwave energy is converted into heat energy [21]. In this study, microwave-assisted extraction (MAE) was used in the sample preparation. Through microwave enhancement, the sample extraction efficiency and recovery were much better than conventional heating processes, so its application in the extraction and separation of natural products increased rapidly. The diffusion capacities of target components may be improved by the increase of solvent temperature under microwave heating [22]. Compared with conventional methods, such as liquid-liquid extraction, Soxhlet extraction, and reflux extraction, MAE has the advantages with shorter extracting time, less solvent usage, higher extraction rate, and superior sample purity at a lower cost [23]. Moreover, MAE has enviable flexibility in the choice of container, sample size, temperature, pressure, and solvent volume. This flexibility makes this extraction method suitable for high-throughput sample preparation and routine analysis.

## 2. Materials and Methods

### 2.1. Chemicals and Reagents

The reagents of methanol, n-hexane, and acetone were obtained from Fisher (Shanghai, China), Tianjin Concord Technology Co., Ltd. (Tianjin, China), and Rilianlong Bohua Pharmaceutical Chemistry Co., Ltd. (Tianjin, China), respectively. Cyclohexane, toluene, chlorobenzene, and styrene were purchased from Sinopsin Group Chemical Reagent Co., Ltd. (Shanghai, China). Benzene was purchased from Aladdin (Shanghai, China). Distilled water was provided by Wahaha Co., Ltd. (Shenyang, China).

Seven brands of soy source samples were purchased in supermarkets with different packagings, such as glass bottles, plastic bags, and plastic bottles.

### 2.2. Instruments and Conditions

The microwave extraction was performed with a Multiwave^TM^ PRO microwave digester from Anton Paar (Graz, Austria).

The GC-MS analysis was performed using an Agilent 6890N-5973 system equipped with an Agilent 122–1334: DB-624 MS capillary column (30 m × 250 *μ*m, 1.4 *μ*m) with a headspace sampler. The sample injection was carried out with a split ratio of 10 : 1. The oven temperature started at 50°C, followed by an increase of 20°C/min up to 170°C. The final temperature was increased up to 230°C, maintaining for 3 min. The ion source and inlet temperatures were controlled at 230°C and 240°C, respectively. The detector was operated in multiple reaction monitoring (MRM) detection mode.

For headspace sampler settings, the heating box temperature was kept at 90°C, the quantitative ring temperature was 190°C, transmission line temperature was 230°C, and balance time was 30 min.

**Figure 1:**
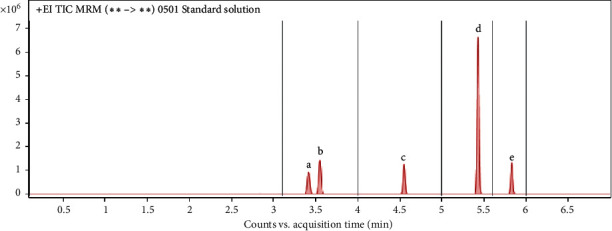
Total ion chromatogram of five volatile organic compounds of soy sauces. a: cyclohexane, b: benzene, c: toluene, d: chlorobenzene, e: styrene.

The mass spectrometric parameters of the five samples are shown in Table 1, and the chromatogram is shown in Figure 1.

### 2.3. Preparation of Sample and Standard Solutions

#### 2.3.1. Preparation of Standard Solutions

We accurately weigh the reference substances of cyclohexane (50 mg), benzene (25 mg), toluene (25 mg), chlorobenzene (25 mg), and styrene (25 mg), then placed them in a 25 mL volumetric flask, and dissolved and diluted them to the mark with methanol, to obtain a cyclohexane concentration of 2 mg/mL and other standard stock solutions with a concentration of 1 mg/mL. We precisely measure 1.0 mL of each of the above reference substance solutions and placed them in a 50 mL volumetric flask, and dissolved and diluted them with methanol to obtain a mixed reference substance intermediate solution, where the concentration of cyclohexane was 40 *μ*g/mL, and the other reference substances were 20 *μ*g/mL. All solutions were stored at 4°C for subsequent experiments.

#### 2.3.2. Preparation of Samples

**Figure 2:**
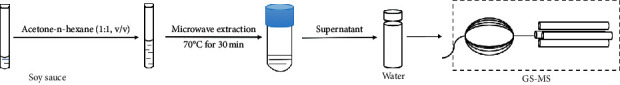
The procedure of sample preparation.

The procedure of sample preparation is shown in Figure 2. 1 mL soy sauce was transferred into a microwave extraction tank, followed by mixing with 8 mL acetone-n-hexane (1 : 1, v/v), and then heated in a microwave at 70°C for 30 min. We took a headspace sample bottle containing 1.8 mL of purified water and injected 200 *μ*L of sample extract for determination.

#### 2.3.3. Method Validation

This experiment validated the reliability of the method from the aspects of linearity, sensitivity, precision, repeatability, and accuracy.

The linearity of the method was investigated in the concentration range of 5–400 ng/mL for reference substance intermediate solution diluted with methanol to a gradient reference solution. We precisely measured 1, 4, 16, 32, 48, 64, and 80 *μ*L of the mixed reference substance intermediate solution, diluted them to 4 mL and 8 ml with methanol, respectively, and filtrated the 0.22 *μ*m filter membrane to save the sample for testing. The limit of detection (LOD) and limit of quantitation (LOQ) of the instrument denoted the lowest detectable and quantitative amount of target analytes in this instrument. We diluted the standard reference solution with methanol to 1 ng/mL and quantified six samples including the blank sample (methanol). We referred to the blank sample spectrum, and the corresponding concentration of the standard reference substance at signal-to-noise (S/N) = 3 : 1 and 10 : 1 was regarded as LOD and LOQ.

Method precision and repeatability were validated from intraday and interday analysis of spiked samples. A series of standard reference solutions were prepared, including 100, 200, and 300 ng/ml cyclohexane, and 200, 400, and 600 ng/ml benzene, toluene, chlorobenzene, and styrene. Two identical sample solutions were prepared in the same way, one of which was added to an equal volume of the standard reference solution. According to the above method to process, two sample solutions were measured in triplicate every day for three consecutive days.

The accuracy of the method was determined by the spiked recovery of each target analyte. And the spiked recovery was calculated as follows:

(1) $$\begin{array}{c} R\left(\%\right)=\frac{R_{1}-R_{0}}{R_{0}}, \end{array}$$

where *R* was the recovery of the method, *R*_0_ was the recovery in the sample solution, and *R*_1_ was the recovery of the spiked sample solution. The experiment subtracted the recovery in the sample solution in order to remove the influence of background on the content determination.

## 3. Results and Discussion

### 3.1. Optimization of Microwave Extraction Parameters

Before method validation, given to the process of the ME, various parameters that may affect the efficiency of the samples were studied including extraction solvent, extraction temperature, and extraction time.

#### 3.1.1. Optimization of Extraction Solvent

**Figure 3:**
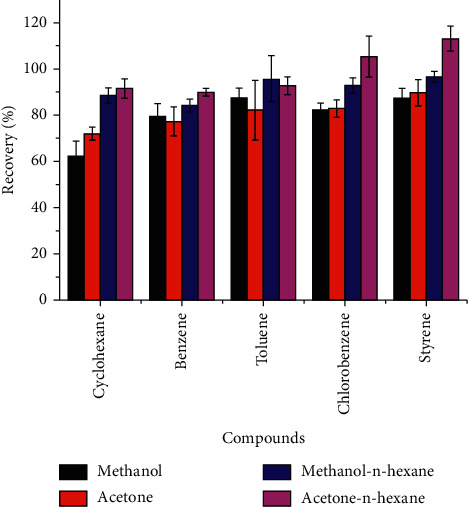
Effect of the type of extraction solvents on extraction efficiency of soy sauces.

Extraction solvents must not only have a certain degree of polar in the ME, to heat samples internally by absorbing energy, but also have a strong dissolving ability to dissolve samples entirely. At the same time, we should also consider the boiling point of the solvents to eliminate the influence of residual solvents on the determination [24]. In this study, based on these considerations, four different polar solvents, methanol, acetone, methanol-n-hexane (1 : 1, v/v), and acetone-n-hexane (1 : 1, v/v), were evaluated, respectively. The other variables were fixed as follows: volume of sample at 1.0 mL, the temperature at 70°C, the volume of solvent at 8 mL, and time of extraction at 30 min. The recoveries of the four solvents are shown in Figure 3. According to the results, five volatile organic compounds can be extracted from the four solvents and the recoveries are 60% above. It is worth mentioning that acetone-n-hexane showed excellent recoveries of five volatile compounds in the microwave extraction, in which cyclohexane, benzene, and toluene were no less than 90%, while chlorobenzene and styrene were close to 110%.

#### 3.1.2. Optimization of Extraction Temperature

**Figure 4:**
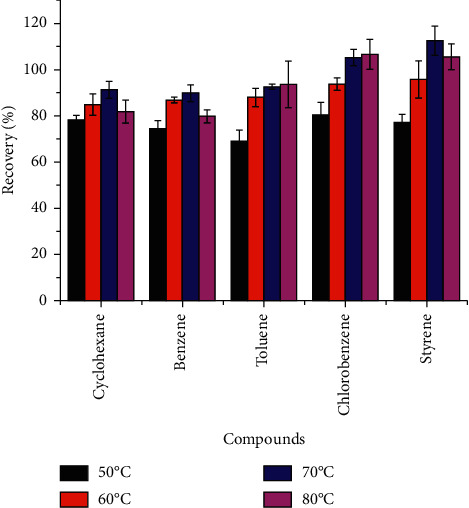
Effect of extraction temperature on extraction efficiency of soy sauces.

In a microwave-sealed container, because the internal pressure reaches a dozen that of the atmosphere, the boiling point of the solvent is higher than that under normal pressure. Therefore, the use of microwave extraction could obtain a temperature that cannot be reached with the same solvent under normal pressure. The higher temperature could improve the extraction efficiency and without altering the chemical structure of the target analytes [25]. The effect of different extraction temperatures on the extraction recoveries was investigated in the range of 50°C–80°C for the same condition. The other variables were fixed as follows: volume of sample at 1.0 mL, the volume of solvent at 8 mL, type of solvent = acetone-n-hexane (1 : 1, v/v), and time of extraction at 30 min. The curves are shown in Figure 4. The results showed that the recoveries of the five volatile organic compounds increased with the increasing temperature within a certain range. However, when it was 80°C, the recovery curve showed a slightly decreasing trend in cyclohexane, benzene, and styrene. Considering the above situation, 70°C was the optimum temperature for microwave extraction.

#### 3.1.3. Optimization of Extraction Time

**Figure 5:**
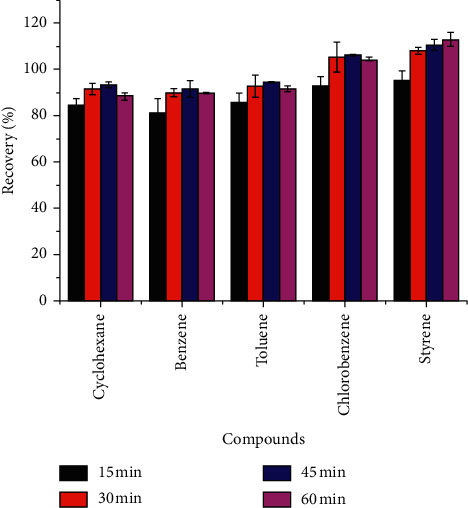
Effect of extraction time on extraction efficiency of soy sauces.

Proper extraction time can improve the efficiency of the method. The extraction time of 15, 30, 45, and 60 min was investigated, and the results are shown in Figure 5. The other variables were fixed as follows: volume of sample at 1.0 mL, the volume of solvent at 8 mL, and type of solvent = acetone-n-hexane (1 : 1, v/v). There was a significant increase in peak areas from 15 min to 45 min, but after 45 min, the extraction efficiency decreased. Due to the cost of heating and the recoveries of the five volatile organic compounds, the final decision was made to select an extraction time of 30 min. The results showed that the material in the microwave-sealed container reached equilibrium in 30 min and indicated that ME had a better extraction effect and took a comparatively shorter time, compared to the previously mentioned conventional methods that usually required hundreds of minutes [26–28].

### 3.2. Method Validation

#### 3.2.1. Linearity and Range

The linearity of each volatile organic compound was investigated by analyzing standard solutions at six concentrations in the range of 5–400 ng/mL. The correlation coefficient in each case was higher than 0.99, which demonstrated that the linear relationship was good. The results are shown in Table 2.

#### 3.2.2. Limit of Detection (LOD) and Limit of Quantization (LOQ)

The LODs and LOQs of the five volatile organic compounds were determined based on S/N at about 3 and 10, respectively. The LODs were 0.25–1.00 ng/mL, and the LODs were 0.50–2.00 ng/mL, which showed that the sensitivity of the instrument was satisfactory. The results can be found in Table 2.

#### 3.2.3. Accuracy and Precision

The accuracy and precision of the method were assessed by spike and recovery experiments, and the results are shown in Table 3. From the results, the recoveries of analytes were 80.86%–105.71%. Among them, the average recovery of benzene was close to 95%, while chlorobenzene, toluene, cyclohexane, and styrene were also 90% around, which proved that the method had good accuracy of the five volatile organic compounds. The precision and repeatability of this method were evaluated by the determination of RSD of target analytes in the samples. The RSD values of intraday and interday precision are lower than 12.1% and 12.5%, respectively, which indicated that the method meets the requirements of soy sauce analysis and testing.

### 3.3. Sample Analysis

The soy sauce samples were purchased from local supermarkets and stored at room temperature. According to the sample preparation method of the section mentioned above, different brands of soy sauce were extracted and analyzed. The results showed that cyclohexane, toluene, chlorobenzene, and styrene were not detected. In four samples with a plastic packing, benzene was detected at a level of 156.42, 129.69, 182.16, and 229.68 *μ*g/mL, respectively.

### 3.4. Comparison to Reported Methods

The comparison between the previously reported method and our proposed method for analyzing cyclohexane, benzene, toluene, chlorobenzene, and styrene in different products is listed in Table 4. Identification of the five harmful volatiles in water, ambient air, fruit, pesticide, and food samples was reported, but the sample preparation using microwave extraction has rarely been reported for the analysis of volatiles in soy sauce. For the reported HS-GC-MS method without the procedure of sample preparation, the LOD of benzene and toluene in water ranged from 0 to 17 ng/mL and from 0 to 35 ng/mL [31]. Raquel et al. applied the distillation and isotope dilution to the determination of benzene in food, with a LOD of 0.75–2.0 ng mL^−1^ and time for preparation sample of 72 min [35]. At present, compared with a large number of studies on benzene and toluene in various substrates, there are fewer studies on the determination and detection methods of chlorobenzene and cyclohexane, which are also common harmful volatiles. In this paper, the ME–HS–GC-MS combined technology is used to simultaneously determine the content of harmful volatile substances in soy sauce, including chlorobenzene and cyclohexane.

## 4. Conclusions

In this study, a simple and effective method, MAE-GC-MS, was developed for the determination of possible plastic packaging originated contamination in soy sauce: cyclohexane, benzene, toluene, chlorobenzene, and styrene. At the same time, the operating parameters of ME are optimized. This method has the advantages of simple operation, high recovery, and good reproducibility. It provided a reliable method for further research on food safety and quality control.

## Figures and Tables

**Table 1:** MRM parameters of the five volatile organic compounds to be measured.

Compounds	Ion pair	Collision energy (eV)
Cyclohexane	84⟶69	5
	84⟶56	5
Benzene	78⟶51	30
	78⟶39	30
Toluene	91⟶65	20
	91⟶39	20
Chlorobenzene	112⟶77	30
	112⟶51	30
Styrene	104⟶78	30
	104⟶51	30

**Table 2:** Linear range and sensitivity of five volatile organic compounds.

Compounds	Linear equation	*R* ^2^	Linear range/(ng·mL^−1^)	LOD/(ng·mL^−1^)	LOQ/(ng·mL^−1^)
Cyclohexane	*y* = 1.912 × 104*x*–1.060 × 105	0.9934	5–400	1.00	2.00
Benzene	*y* = 2.501 × 104*x*–3.024 × 104	0.9954	5–400	0.50	1.00
Toluene	*y* = 2.964 × 104*x*–2.243 × 105	0.9943	5–400	0.50	1.00
Chlorobenzene	*y* = 9.867 × 104*x*–7.485 × 103	0.9957	5–400	0.25	0.50
Styrene	*y* = 3.668 × 104*x*–6.008 × 105	0.9916	5–400	0.50	1.00

**Table 3:** Accuracy and precision of five volatile organic compounds.

Compounds	Concentrations (ng·mL^−1^)	Intraday						Interday (%)	RSD (%)
		Day 1 (%)	RSD (%)	Day 2 (%)	RSD (%)	Day 3 (%)	RSD (%)		
Cyclohexane	200	90.84	8.2	91.67	12.1	81.94	5.2	88.15	6.1
	400	95.77	2.8	91.50	10.1	86.58	3.6	91.28	5.0
	600	85.28	3.9	96.86	7.5	105.71	4.8	95.95	10.7
Benzene	100	93.24	6.6	94.80	8.8	87.26	4.5	91.77	4.3
	200	97.36	2.6	95.76	8.1	90.60	2.6	94.57	3.7
	300	87.39	3.3	97.12	6.7	104.55	4.5	96.35	8.9
Toluene	100	89.26	7.6	90.65	8.5	85.91	5.2	88.61	2.7
	200	93.76	3.0	91.65	9.4	89.31	2.8	91.57	2.4
	300	83.87	4.8	92.68	7.7	103.83	5.4	93.46	10.7
Chlorobenzene	100	89.71	7.4	92.38	8.2	87.39	4.8	89.83	2.8
	200	93.72	2.8	92.93	9.0	90.78	2.7	92.48	1.6
	300	83.50	4.5	92.59	7.5	103.45	5.5	93.18	10.7
Styrene	100	85.71	9.5	85.82	9.3	84.33	6.2	85.29	1.0
	200	91.15	3.1	89.44	10.3	90.56	3.0	90.38	1.0
	300	80.86	4.8	90.30	8.4	103.70	5.9	91.62	12.5

**Table 4:** Comparison of the method proposed with the method developed earlier in the analysis of volatiles.

Ref.	Analyte	Sample type	Extraction and determination method	Preparation sample time (min)	Linear range (ng/mL)	LOD (ng/mL)	RSD (%)
[29]	Benzene	Pesticide	HS-GC-MS	—	3.2–16.0	—	0.9–1.9
	Toluene				3.6–16.0		4.9–8.1
[30]	Toluene	Fruit	HS-SPME-GC-MS	33	0.011–2.91	0.011	12.5–13
	Styrene				0.074–8.93	0.025	10.9–11.3
[31]	Benzene	Water	HS-GC-MS	—	0–35.0	2	5.5–5.6
	Toluene				0–17.0		7.3–12
[32]	Benzene	Olive oil	LLE–HS–GC-MS	30	1.5–200	0.43	3.3–4.0
	Toluene					0.42	2.8–3.6
	Styrene				1–200	0.38	1.9–2.6
[33]	Benzene	Ambient air	TD-GS-MS/MS	31	1–100	0.004	1–14
	Toluene					0.0004	10–12
	Styrene					0.004	10–16
[34]	Benzene	Water	SPE-GC	35	8770–35080	350–372	6.2–9.8
	Toluene				8670–34080	167–234	14.3–17.4
[35]	Benzene	Food	Distillation and isotope				
Dilution-HS-GC/MS	72	0–21	0.75–2.0	3.7–8.7			
[36]	Benzene	Soil	QuEChERS-GC-MS	21615	50–500	15	2.8–6.1
	Toluene					0.4	1.3–2.7
This study	Cyclohexane	Soy sauce	ME-HS-GC-MS	30	5–400	1.00	5.0–10.7
	Benzene					0.50	3.7–8.9
	Toluene					0.50	2.4–10.7
	Chlorobenzene					0.25	1.6–10.7
	Styrene					0.50	1.0–12.5

## Data Availability

All data generated or analyzed during this study are included within this published article.

## References

[1] Gao X., Cui C., Ren J., Zhao H., Zhao Q., Zhao M. (2011). Changes in the chemical composition of traditional Chinese-type soy sauce at different stages of manufacture and its relation to taste. *International Journal of Food Science & Technology*.

[2] Mejia L. A., Bower A. M. (2015). The global regulatory landscape regarding micronutrient fortification of condiments and seasonings. *Annals of the New York Academy of Sciences*.

[3] Zhao H., Wang Y., Zou Y., Zhao M. (2013). Natural occurrence of deoxynivalenol in soy sauces consumed in China. *Food Control*.

[4] Sun S. Y., Jiang W. G., Zhao Y. P. (2010). Profile of volatile compounds in 12 Chinese soy sauces produced by a high-salt-diluted state fermentation. *Journal of the Institute of Brewing*.

[5] Gao X.-L., Cui C., Zhao H.-F., Zhao M.-M., Yang L., Ren J.-Y. (2010). Changes in volatile aroma compounds of traditional Chinese-type soy sauce during moromi fermentation and heat treatment. *Food Science and Biotechnology*.

[6] Meng Q., Kitagawa R., Imamura M., Katayama H., Obata A., Sugawara E. (2017). Contribution of 2-methyl-3-furanthiol to the cooked meat-like aroma of fermented soy sauce. *Bioscience, Biotechnology, and Biochemistry*.

[7] Meng Q., Imamura M., Katayama H., Obata A., Sugawara E. (2017). Key compounds contributing to the fruity aroma characterization in Japanese raw soy sauce. *Bioscience, Biotechnology, and Biochemistry*.

[8] Li Y., Zhao H., Zhao M. (2010). Relationships between antioxidant activity and quality indices of soy sauce: an application of multivariate analysis. *International Journal of Food Science and Technology*.

[9] Wampler T. P., Marsili R. (2017). Analysis of food volatiles using headspace-gas chromatographic techniques. *Techniques for Analyzing Food Aroma*.

[10] Kim M. K., Jang H. W., Lee K.-G. (2018). Sensory and instrumental volatile flavor analysis of commercial orange juices prepared by different processing methods. *Food Chemistry*.

[11] Wanakhachornkrai P., Lersiri S. (2003). Comparison of determination method for volatile compounds in Thai soy sauce. *Food Chemistry*.

[12] Avsar Y. K., Karagul-Yuceer Y., Drake M. A., Singh T. K., Yoon Y., Cadwallader K. R. (2004). Characterization of nutty flavor in Cheddar cheese. *Journal of Dairy Science*.

[13] Chen F., Zu Y., Yang L. (2015). A novel approach for isolation of essential oil from fresh leaves of Magnolia sieboldii using microwave-assisted simultaneous distillation and extraction. *Separation and Purification Technology*.

[14] Lee S. M., Seo B. C., Kim Y. S. (2006). Volatile organic compounds in fermented and acid-hydrolyzed soy sauces. *Journal of Food Science*.

[15] Lee M.-R., Chiu T.-C., Dou J. (2007). Determination of 1,3-dichloro-2-propanol and 3-chloro-1,2-propandiol in soy sauce by headspace derivatization solid-phase microextraction combined with gas chromatography-mass spectrometry. *Analytica Chimica Acta*.

[16] Feng Y., Cai Y., Su G., Zhao H., Wang C., Zhao M. (2014). Evaluation of aroma differences between high-salt liquid-state fermentation and low-salt solid-state fermentation soy sauces from China. *Food Chemistry*.

[17] Balasubramanian S., Panigrahi S. (2011). Solid-phase microextraction (SPME) techniques for quality characterization of food products: a review. *Food and Bioprocess Technology*.

[18] Abdulra’Uf L. B., Tan G. H. (2015). Chemometric approach to the optimization of HS-SPME/GC–MS for the determination of multiclass pesticide residues in fruits and vegetables. *Food Chemistry*.

[19] Wang X., Fan W., Xu Y. (2014). Comparison on aroma compounds in Chinese soy sauce and strong aroma type liquors by gas chromatography-olfactometry, chemical quantitative and odor activity values analysis. *European Food Research and Technology*.

[20] Ping C. Y., Kei C. T., Yin C. H. (2019). Optimization of a headspace solid-phase microextraction method to quantify volatile compounds in plain sufu, and application of the method in sample discrimination. *Food Chemistry*.

[21] Delazar A., Nahar L., Hamedeyazdan S., Sarker S. D. (2012). Microwave-assisted extraction in natural products isolation. *Methods in Molecular Biology*.

[22] Kaderides K., Papaoikonomou L., Serafim M., Goula A. M. (2019). Microwave-assisted extraction of phenolics from pomegranate peels: optimization, kinetics, and comparison with ultrasounds extraction. *Chemical Engineering and Processing-Process Intensification*.

[23] Zheng X., Wang X., Lan Y., Shi J., Liu C. (2010). Application of response surface methodology to optimize microwave-assisted extraction of silymarin from milk thistle seeds. *Separation and Purification Technology*.

[24] Zghaibi N., Omar R., Kamal S., Biak D., Harun R. (2019). Microwave-assisted brine extraction for enhancement of the quantity and quality of lipid production from microalgae nannochloropsis sp. *Molecules*.

[25] Wen L., Zhang Z., Sun D.-W., Sivagnanam S. P., Tiwari B. K. (2020). Combination of emerging technologies for the extraction of bioactive compounds. *Critical Reviews in Food Science and Nutrition*.

[26] Hirondart M., Rombaut N., Fabiano-Tixier A. S., Bily A., Chemat F. (2020). Comparison between pressurized liquid extraction and conventional soxhlet extraction for rosemary antioxidants, yield, composition, and environmental footprint. *Foods*.

[27] Álvarez-Muñoz D., Sáez M., Gómez-Parra A., González-Mazo E., González-Mazo E. (2004). New extraction method for the analysis of linear alkylbenzene sulfonates in marine organisms. *Journal of Chromatography A*.

[28] George M. J., Sichilongo K. F., Ramabulana T., Madala N. E., Dubery I. A. (2019). Comparison of Soxhlet and reflux techniques for extraction and characterisation of potential endocrine-disrupting compounds from solid waste dumpsite soil. *Environmental Monitoring and Assessment*.

[29] Lidong C., Jiang H., Yang J., Fan L., Li F., Huang Q. (2013). Simultaneous determination of benzene and toluene in pesticide emulsifiable concentrate by headspace GC-MS. *Journal of Analytical Methods in Chemistry*.

[30] Bosse Nee Danz R., Wirth M., Konstanz A., Becker T., Weiss J., Gibis M. (2017). Determination of volatile marker compounds in raw ham using headspace-trap gas chromatography. *Food Chem*.

[31] Pérez Pavón J. L., del Nogal Sánchez M., Fernández Laespada M. E., Moreno Cordero B. (2007). Simultaneous determination of gasoline oxygenates and benzene, toluene, ethylbenzene and xylene in water samples using headspace-programmed temperature vaporization-fast gas chromatography-mass spectrometry. *Journal of Chromatography A*.

[32] Carrillo-Carrión C., Lucena R., Cárdenas S., Valcárcel M. (2007). Liquid-liquid extraction/headspace/gas chromatographic/mass spectrometric determination of benzene, toluene, ethylbenzene, (o-, m- and p-) xylene and styrene in olive oil using surfactant-coated carbon nanotubes as extractant. *Journal of Chromatography A*.

[33] Feng L., Hu X., Yu X., Zhang W. (2016). Determination of volatile organic compounds in ambient air by thermal desorption-gas chromatography-triple quadrupole tandem mass spectrometry. *Chinese Journal of Chromatography*.

[34] Mottaleb M. A., Abedin M. Z., Islam M. S. (2003). Determination of benzene, toluene, ethylbenzene and xylene in river water by solid-phase extraction and gas chromatography. *Analytical Science*.

[35] Vinci R. M., De Meulenaer B., De Schaetzen T., Van Overmeire I., De Beer J., Van Loco J. (2010). Determination of benzene in different food matrices by distillation and isotope dilution HS-GC/MS. *Analytical Chimica Acta*.

[36] Pinto C. G., Martín S. H., Pérez Pavón J. L., Bernardo M. C. (2011). A simplified Quick, Easy, Cheap, Effective, Rugged and Safe approach for the determination of trihalomethanes and benzene, toluene, ethylbenzene and xylenes in soil matrices by fast gas chromatography with mass spectrometry detection. *Analytical Chimica Acta*.

